# Genomes of Alphanucleorhabdovirus Physostegiae Isolates from Two Different Cultivar Groups of *Solanum melongena*

**DOI:** 10.3390/v16101538

**Published:** 2024-09-28

**Authors:** Nikita Gryzunov, Sergey Yu. Morozov, Tatiana Suprunova, Viktoriya Samarskaya, Nadezhda Spechenkova, Sofiya Yakunina, Natalia O. Kalinina, Michael Taliansky

**Affiliations:** 1 Shemyakin-Ovchinnikov Institute of Bioorganic Chemistry of the Russian Academy of Sciences, 117997 Moscow, Russia; nikgr@ibch.ru (N.G.); v.sam@ibch.ru (V.S.);; 2 Faculty of Bioengineering and Bioinformatics, Lomonosov Moscow State University, 119234 Moscow, Russia; 3 Belozersky Institute of Physico-Chemical Biology, Lomonosov Moscow State University, 119991 Moscow, Russia; morozov@belozersky.msu.ru; 4 Doka-Gene Technologies Ltd., 141880 Rogachevo, Russia; 5 The James Hutton Institute, Invergowrie, Dundee DD2 5DA, UK

**Keywords:** nucleorhabdoviruses, *Physostegia chlorotic mottle virus* (PhCMoV), PhCMoV European isolates, Russian PhCMoV isolates, high-throughput sequencing

## Abstract

Plant rhabdoviruses cause considerable economic losses and are a threat to the agriculture of *Solanaceae* plants. Two novel virus isolates belonging to the family *Rhabdoviridae* are identified by high-throughput sequencing (HTS) in Russian eggplant cultivars grown in the Volga river delta region for the first time. The phylogenetic inference of L protein (polymerase) shows that these virus isolates belong to Alphanucleorhabdovirus physostegia (*Physostegia chlorotic mottle virus*—PhCMoV), and their minus-sense RNA genomes have the typical gene order 3′-nucleocapsid (N)—X protein (X)—phosphoprotein (P)—Y protein (Y)—matrix protein (M)—glycoprotein (G)—polymerase (L)-5′ observed in some plant-infecting alphanucleorhabdoviruses. One of the PhCMoV isolates from the eggplant cultivar Almaz is genetically very similar to the Russian PhCMoV isolate from tomato and grouped in a subclade together with four isolates from Belgium, Germany, the Netherlands, and France. However, another eggplant-infecting isolate from the Russian cultivar Boggart is the most divergent compared with the other 45 virus genomes of European PhCMoV isolates. Thus, our comparative analysis reveals that two virus isolates from Russia may either share a close evolutionary relationship with European isolates or significantly diverge from all known virus isolates. The potential to use the protein sequence comparative analysis of accessory polypeptides, along with the early developed strategy of the nucleotide sequence comparison of the RNA genomes, is shown.

## 1. Introduction

Plant RNA viruses have an outstanding ability to achieve a high evolution rate due to fast replication and rapid accumulation of mutations since their RNA replicases lack proofreading activity [1,2]. The genetic variations produced by mutation and recombination processes are known to be under pressure of natural selection resulting from the competition between genetic variants influencing important aspects of the virus life cycle [3,4]. As a result, a limited number of variants with high reproductive fitness will pass to the next generations. In limited virus populations, genetic drift also influences the frequency of genetic variants by random chance [2,3,4]. Gene flow favors genetic uniformity between virus populations due to the selection process, whereas the restricted migration of genetic variants because of geographic/biological factors and genetic drift leads to enhanced genetic differentiation among populations. In general, the characterization of the genetic variability among virus populations is important for developing accurate detection and diagnostic tools and implementing efficient disease control strategies [4,5].

Eggplant (*Solanum melongena* L.) is an economically important vegetable crop worldwide. Eggplant ranks as the third most significant crop within the *Solanaceae* family after potatoes and tomatoes [6,7]. It is known that eggplant varieties can be infected by fungi, bacteria, and viruses that may cause important diseases with a negative impact on production and fruit quality [8,9,10]. In particular, there are two nucleorhabdoviruses named Alphanucleorhabdovirus melongenae [*Eggplant mottled dwarf virus* (EMDV)] and Alphanucleorhabdovirus physostegiae [*Physostegia chlorotic mottle virus* (PhCMoV)] that have negative effects on crop production [11,12,13]. These viruses cause deformed leaves of varying intensity, which have chlorotic to yellow discolorations of the veins. Foliar symptoms may be accompanied by mild-to-severe plant stunting and a lack of fruit. The fruits, when present, are small and often deformed [11,12,13]. These two virus species positioned in the genus *Alphanucleorhabdovirus* (family *Rhabdoviridae*, order *Mononegavirales*) encode five conserved proteins in the negative-sense RNA genome 3′-to-5′ direction [nucleocapsid (N), phosphoprotein (P), matrix (M), glycoprotein (G), and the polymerase (L)], like other rhabdoviruses. Furthermore, both EMDV and PhCMoV and three other nucleorhabdoviruses contain an accessory Y gene inserted between P and M and encoding the virus movement protein, as well as an additional auxiliary one (X gene) located between N and P, giving rise to an acidic protein with a currently unknown function [11,14,15,16,17,18]. All nucleorhabdovirus proteins are at least partly localized in the nuclei of host cells [19]. In the minus-sense rhabdovirus genomic RNA, these seven genes are separated by intergenic untranslated regions (UTRs), which, along with the 3′ leader and 5′ trailer sequences, are essential for the replication and transcription of the viral RNA. The intergenic UTRs contain termination and promoter signals, which are required during transcription to produce monocistronic mRNAs [11,13,15,16,17,18].

RNA viruses, such as nucleorhabdoviruses, exist as quasi-species, with the emergence of new strains and species being a continuous process [20]. EMDV and PhCMoV strain populations are widespread among crops (including eggplant) in the Mediterranean basin and have also been detected in many other European countries [11,12,13,21]. In 2022, to allow for efficient comparisons of epidemiologically important PhCMoV lineages, researchers from eight European laboratories proposed that “the current situation requires rapid characterization and a common response from European countries” to detect PhCMoV in Europe over a wide range of economically important plant crops [11]. However, currently, there is only a single report showing the presence of PhCMoV in the tomato sample from Russia [11]. Thus, data on the possible infection of eggplants by nucleorhabdoviruses in Russia are practically absent.

In the last few decades, in addition to well-established serological methods (mainly enzyme-linked immunosorbent assay, ELISA), molecular techniques (hybridization and DNA amplification, particularly PCR) have been developed for the detection of plant viruses [5,22]. High-throughput sequencing (HTS) technologies have resulted in enormous progress in plant virus diagnosis and identification [16,23,24]. HTS is not dependent on previous knowledge of viral sequences in a plant probe and results in the rapid diagnosis of all viruses (even unknown) present in plants (both symptomatic and asymptomatic), although often it does not show a direct association between the symptomatic disease and the detected viruses [25,26]. In this paper, we used HTS to detect nucleorhabdoviruses infecting eggplants in Russia (the Volga river delta region). The first report on the plant rhabdoviruses (represented by two isolates of PhCMoV) in Russian cultivars of *Solanum melongena* is presented.

## 2. Materials and Methods

Samples of leaf material from two eggplant cultivar groups, Boggart (Bog) and Almaz (Alm), were obtained on 3 August 2023 from asymptomatic field-grown plants in the Astrakhan region near the Volga river delta in Russia from true leaves at stages 4–5. Leaves were collected in a fixative buffer (Fixator IntactRNA (Evrogen, No. BC031, Moscow, Russia), 20–22 plants per cultivar. The isolation of total RNA was performed using TRIzol, yielding two tubes of RNA preparations per sample, which were dissolved in nuclease-free water (NFW, Thermo Fisher Scientific, Waltham, MA, USA). RNA samples were treated with DNase I (RNase-free, 50 U/µL, Thermo #EN0523, a new batch from BioInnLabs, Rostov-on-Don, Russia) at a ratio of 1 unit per 1 µg of RNA. Moreover, 1 μL of RiboCare RNase Inhibitor was added to each sample (40 e.a./μL, EK005S, Eurogen, Moscow, Russia). The mixture was incubated at 37 °C for 30 min before being resuspended with TRIzol-chloroform without temperature termination. The RNA quantity and quality were determined using gel electrophoresis and a NanoDrop spectrophotometer (Thermo Fisher Scientific, Waltham, MA, USA). Total RNA libraries were constructed using TruSeq Stranded Total RNA with a Ribo-Zero Plant kit (Illumina, San Diego, CA, USA), which enables rRNA depletion. Libraries were sequenced on Illumina NovaSeq 6000 (2 × 101 bp). Reads were demultiplexed using Illumina bcl2fastq v2.20. Adapter removal was achieved through the use of Skewer v0.2.2 [27]. Sequencing, demultiplexing, and adapter removal were performed by CeGaT GmbH (Tuebingen, Germany). The RNA-sequencing libraries without adapters were deposited in the Sequence Read Archive (SRA) under BioProject accession number PRJNA1144656.

Read quality was evaluated with the FastQC v0.11.9 (https://www.bioinformatics.babraham.ac.uk/projects/fastqc/, accessed on 8 June 2024) and SeqKit v2.3.0 [28] tools. A total of 34,178,077 and 26,858,940 paired-end 101 nt reads were obtained for the RU31-Boggart and RU32-Almaz libraries, respectively, via HTS cDNA library construction. These two libraries demonstrated good sequencing quality, with Q30 values ranging from 88.32 to 94.09% across both libraries, and reads from the libraries were normalized using bbnorm.sh v39.01 (BBMap—Bushnell B.—https://sourceforge.net/projects/bbmap/, accessed on 8 June 2024) and then mapped to the Solanum melongena genome assembly GCA_000787875.1 using hisat2 v2.1.0 to remove host reads. Then, *de novo* assembly by Trinity v2.14.0 (standard k-mer length, 25 nt) [29] produced contig sets of 10,836 and 8956 sequences, respectively. Contig taxonomy was determined using BLASTn v2.14.0+ [30], and the ref_viruses_rep_genomes BLAST database (NCBI, Bethesda, MD, USA) was used for annotation. Several contigs were identified as associated with plant viruses. Two contigs (RU31-Bog and RU32-Alm) >13 kb in size revealed similarities to nucleorhabdoviruses (see the Section 3). The remaining contigs showed nearly 99% homology to cucumber mosaic virus RNA1, RNA2, and RNA3. RU31-Bog and RU32-Alm contigs were validated through independent assemblies using rnaSPAdes v3.15.4 [31] and rnaviralSPAdes v3.15.4 [32]. To further validate contigs, the original libraries were mapped to contigs using hisat2 v2.1.0 (https://daehwankimlab.github.io/hisat2/, accessed on 8 June 2024), followed by the removal of reads with mismatches. Both contigs had coverage depths of at least 100 reads throughout most of their length. These two contigs were submitted to GenBank with the accession numbers PQ206275 and PQ206276.

Detailed sequence comparisons were also carried out using the BLAST algorithm (BLASTn and BLASTp) at NCBI. Open reading frames (ORFs) were identified using the ORF Finder programs (https://www.ncbi.nlm.nih.gov/orffinder/, accessed on 8 June 2024). Conserved motif searches were conducted in CDD (http://www.ncbi.nlm.nih.gov/Structure/cdd/wrpsb.cgi/, accessed on 8 June 2024) databases. Phylogenetic analysis was performed with “Phylogeny.fr” (a free, simple-to-use web service dedicated to the reconstruction and analysis of phylogenetic relationships between molecular sequences) by constructing maximum likelihood phylogenetic trees (http://www.phylogeny.fr/simple_phylogeny.cgi/, accessed on 8 June 2024). Bootstrap percentages received from 1000 replications were used.

## 3. Results

### 3.1. Genome Sequences of the Revealed Eggplant Rhabdoviruses

**Figure 1:**
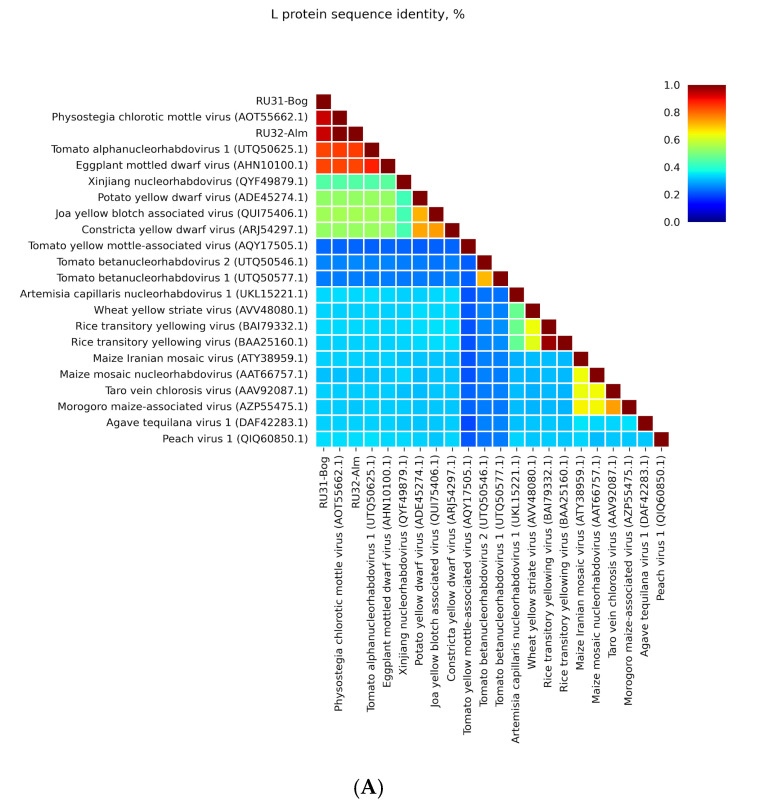

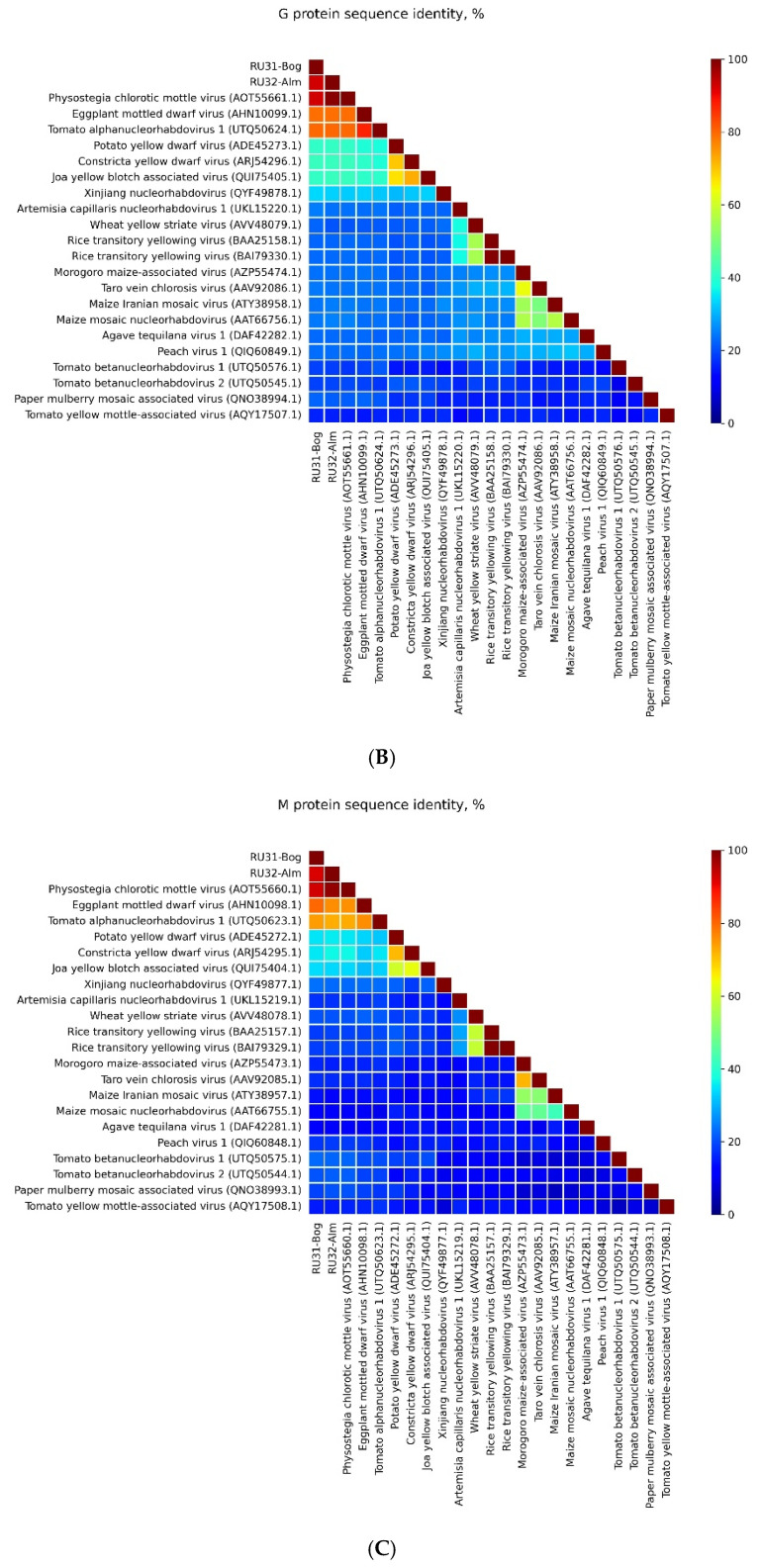

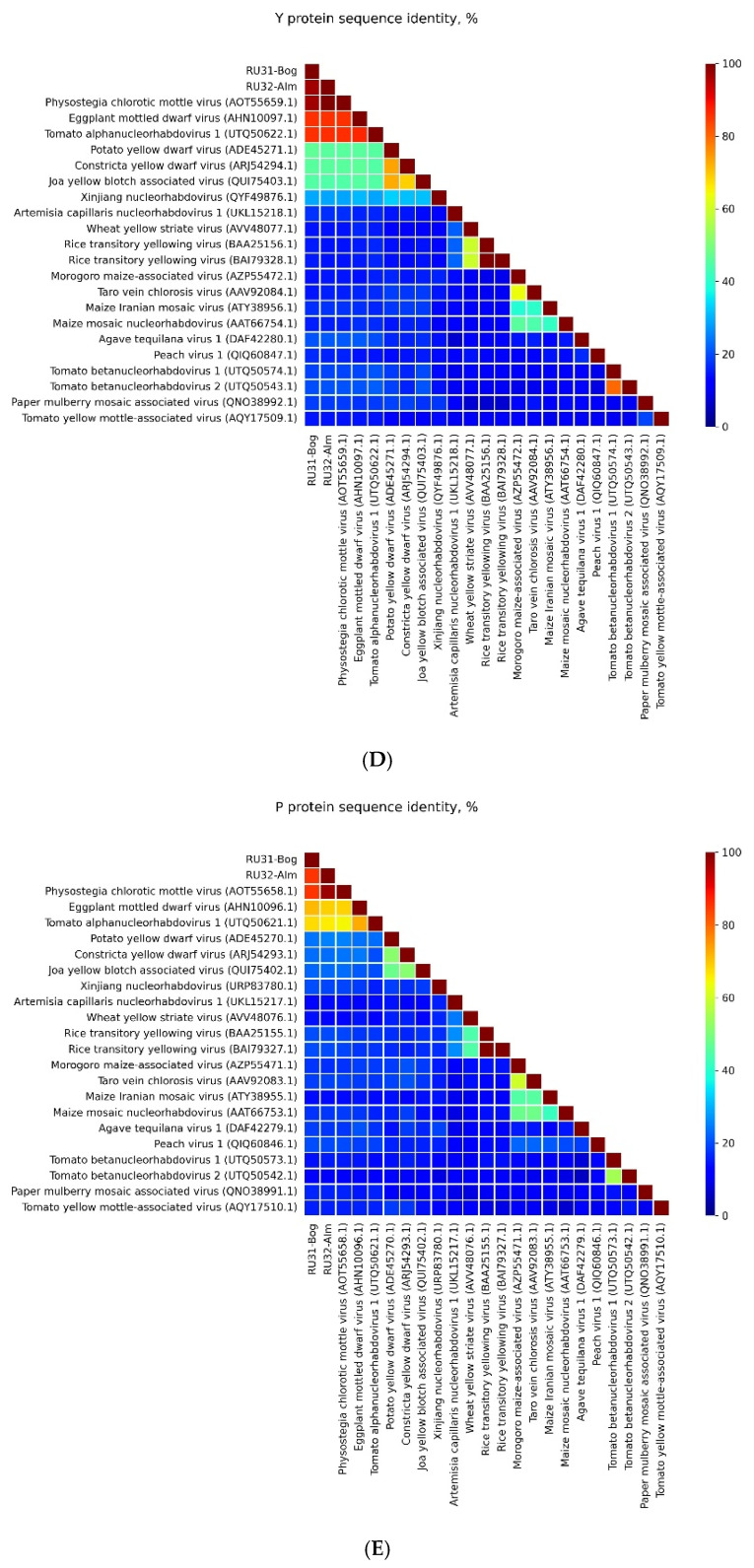

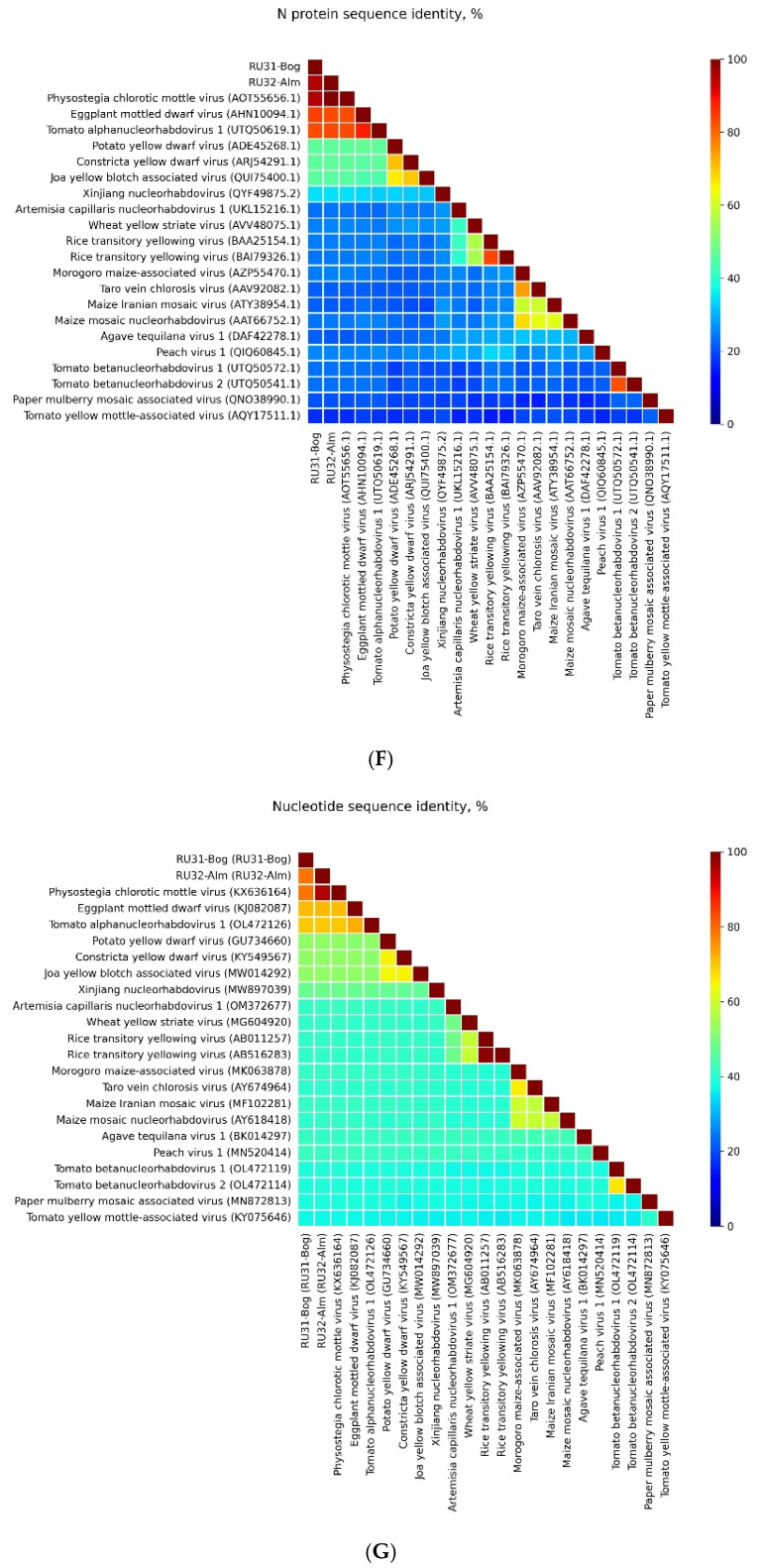
Pairwise identity matrix of the amino acid (**A**–**F**) and nucleotide (**G**) sequences in nucleorhabdovirus proteins and genomes: (**A**) RNA polymerase (L protein); (**B**) G protein; (**C**) M protein; (**D**) Y protein; (**E**) P protein; (**F**) nucleocapsid protein; (**G**) RNA genome sequences.

Our HTS studies of RNA samples isolated from two Russian eggplant cultivars, Boggart (Bog) and Almaz (Alm), have revealed, in each cultivar, long contigs resembling the size of plant rhabdovirus negative-sense RNA genomes. We have named these probable virus genomes RU31-Bog (size 13,197 nts) and RU32-Alm (size 13,307 nts). NCBI BLASTx analysis has unambiguously shown that both contigs encode polymerase proteins highly similar to those of virus species belonging to the genus *Alphanucleorhabdovirus*. Pairwise sequence comparisons at NCBI BLASTp have revealed that proteins G, M, Y, P, and N encoded by both found eggplant viruses are most similar to the corresponding proteins of PhCMoV (identity 92–94%), whereas RNA polymerases (protein L) have smaller similarity to other alphanucleorhabdoviruses, EMDV, and *Tomato alphanucleorhabdovirus 1* (identity near 84%) (Figure 1A–F). The genomic nucleotide sequences of these sequenced isolates have 79% and 82% of their identity matched to most of the other PhCMoV isolates (Figure 1G).

**Figure 2:**
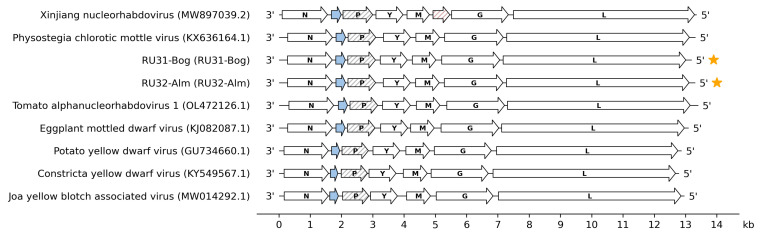
Genome organization in nucleorhabdovirus species. Accessory genes are colored blue (X protein). Hatches: P protein. Two novel isolates (RU31-Bog and RU32-Alm) are marked with orange stars.

These data strongly suggest that the sequenced eggplant viruses represent isolates (strains) of PhCMoV because one of the criteria used to differentiate species within the genus *Alphanucleorhabdovirus* is that the nucleotide sequence identities of entire genomes must be less than 75% [13]. Sequences of RU31-Bog and RU32-Alm have been translated into amino acid sequences of open reading frames (ORFs) via NCBI ORF Finder. Both genomes encode seven genes in the antigenomic sense (Figure 2). These genomic RNAs contain, in the 3′-to-5′ direction, coding regions for the proteins N, X, P, Y, M, G, and L. Their genome organization is similar to those of EMDV, *Potato yellow dwarf virus* (PYDV), *Constricta yellow dwarf virus* (CYDV), and *Joa yellow blotch-associated virus* (JYBaV) but differs from those of other alphanucleorhabdoviruses (Figure 2).

All genes of RU31-Bog and RU32-Alm are separated by conserved gene junction regions with the consensus sequence 3′-AAAUUAUUUUU **GGG** UUG-5′, which represents the polyadenylation signal of the upstream gene, untranscribed G-rich intergenic region (in bold letters), and transcription initiation site for the downstream gene and is similar to those of other alphanucleorhabdoviruses [14,16,18] (Table 1). The alphanucleorhabdovirus genome region containing seven genes is flanked by the so-called 3′ leader and 5′ trailer [17,18].

### 3.2. Placement of RU31-Bog and RU32-Alm in the Phylogenies of the Previously Sequenced PhCMoV Isolates

**Figure 3:**
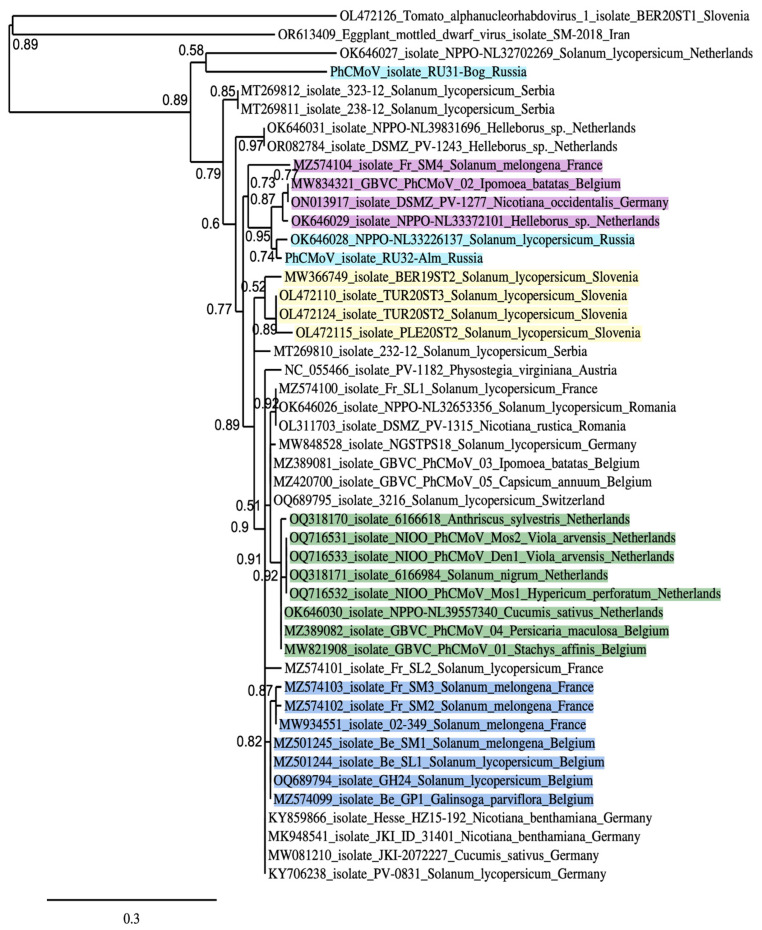
The phylogenies of the 45 PhCMoV strains based on X protein sequences. Accession numbers, host species, and countries of isolation are shown. Russian isolates are shown in blue. The cluster of Slovenian strains is shown in yellow. Strains related to RU32-Alm are shown in pink. The scale bar indicates the number of substitutions per site. The green and light blue colors indicate sequence clusters including isolates from Western Europe.

PhCMoV was first identified in Austria by the HTS method in *Physostegia virginiana* plants collected in 2014 [33]. Later, using HTS allowed for the detection of novel PhCMoV strains in Germany and Serbia on tomatoes [34,35]. Comprehensive comparative population analyses of more than 30 virus strains infecting plant species around Europe have previously been published [11,12]. PhCMoV has also been found to naturally infect 21 plant species across 15 plant families and is transmitted by a polyphagous leafhopper (genus *Anaceratagallia*) representing a natural vector [12]. It was previously revealed that relatively low genetic variability was observed for the genomic sequences (>93% nucleotide identity) in 28 out of 29 isolates [11]. Alternatively, to understand the evolutionary relationships among the 45 PhCMoV isolates in our study, amino acid identities were calculated for X proteins (Figure 3), which are the most divergent among plant nucleorhabdoviruses [11,17,21].

These phylogenetic data show that RU31-Bog is significantly diverged from other PhCMoV strains (Figure 3), like the strain NPPO-NL32702269 isolated from *Solanum lycopersicum* in the Netherlands [11]. Indeed genomic nucleotide sequences of these isolates have 79% and 82% common identity with most other isolates, which in turn show a common identity of 95–98% with each other (Table 2 and Table 3) [11]. RU31-Bog and NPPO-NL32702269 show 80% common identity in pairwise nucleotide sequence comparison. The phylogenetic tree of PhCMoV X proteins contains several well-defined basal clusters (Figure 3). In particular, two Russian isolates, RU32-Alm from eggplant (this paper) and NPPO-NL33226137 from tomato [11], are included in a common cluster with four other strains isolated in France (eggplant), Belgium (*Ipomea batatas*), Germany (*Nicotiana occidentalis*), and the Netherlands (*Helleborus* sp.) (Figure 3). Importantly, the members of this cluster also have highest common nucleotide sequence identity with RU32-Alm among the PhCMoV strains (Table 2), thus showing clear sequence similarity correlation between X protein amino acid sequences and genome nucleotide sequences. One more phylogenetic cluster has been found to contain four Slovenian isolates (Figure 3). The members of this cluster also show the highest common genomic nucleotide sequence identities among PhCMoV strains (Table 3). Since the need to organize PhCMoV strains into different genetic groups or clusters was first recognized in a recent paper when isolates were primarily divided into genogroups based on full-length genome sequences [11], our data further suggest that we should designate genogroups based on the complete X protein amino acid sequences together with the data on genome nucleotide sequences. Importantly, the use of well-conserved amino acid sequences of RNA polymerase (L protein) or nucleocapsid protein, which are universally used as phylogenetic anchors, provides only poorly informative dendrograms with negligible amounts of distinct sequence clusters (Figure S1) due to very high sequence similarity (92–98%) between PhCMoV isolates.

## 4. Discussion

To better understand the genetic diversity and evolution of PhCMoV in Europe, the accumulation of genomic sequences from many diverse isolates is required [11]. Recent data highlight the importance of the geographical location on the virus population evolution of PhCMoV strains [11]. Moreover, this is in line with observations on other plant rhabdoviruses, where the phylogenetic position among strains correlates with geographical localization but not with the host plant [21,36,37]. These observations suggest that certain genetic variations may have occurred during the virus’ geographic spread and then fixed to promote the virus’ viability in specific areas.

Our study of two Russian isolates adds some data on the occurrence and genomic diversity of PhCMoV isolates from a novel geographical location near the border between Europe and Asia. Importantly, Russian isolates (RU31-Bog and RU32-Alm) have been identified in the same relatively small region (the Astrakhan region near the Volga river delta). Nevertheless, when the amino acid sequence identities of the different isolates of PhCMoV were compared in phylogenetic and pairwise analyses, RU31-Bog was the most divergent isolate, having an isolated position in the phylogenetic tree of X proteins (Figure 3) and showing 79% nucleotide sequence identity compared with the other 45 strain genomes. Another most divergent isolate, NPPO-NL32702269, with 81–82% identity has been found in the Netherlands [11]. Both these isolates are quite distant from each other and evidently belong to PhCMoV strains, because one criterion used to differentiate species within the genus *Alphanucleorhabdovirus* is that the nucleotide sequence identity of entire genomes must be less than 75% [13].

Interestingly, RU32-Alm and the Russian isolate NPPO-NL33226137 from tomato are clearly clustered with four other strains isolated in Western Europe (France, Belgium, Germany, and the Netherlands). These data are not correlated with the high impact of the geographical dimension on the genetic evolution of PhCMoV. However, obvious clustering of four PhCMoV strains from Slovenia (Figure 3, Table 3) is in accordance with the proposed significance of geographical location as a determinant of genetic diversity. Revealing another sub-cluster of eight isolates from *Anthriscus sylvestris*, *Viola arvensis*, *Solanum nigrum*, *Hypericum perforatum*, *Cucumis sativus*, *Persicaria maculosa* and *Stachys affinis* found in the neighboring countries (The Netherlands and Belgium) (Figure 3) also supports the significance of a common geographical location for the existence of a close genetic relationship.

Taken together with a previous study [11], our data confirm that our current understanding of the determinants of genetic diversity in virus populations remains partly incomplete, and finding more viral strain genomes in samples of different origins (new location and new host) is important for gaining a better understanding of the genetic diversity and evolution of PhCMoV.

## Figures and Tables

**Table 1:** The genome positions of intergenic junction sequences in PhCMoV isolates sequenced in this paper.

Location of GGG Sequence in Leader and Intercistronic Regions of Genomic RNA	Nucleotide Position of Central G Residue in RU31-Bog *	Nucleotide Position of Central G Residue in RU32-Alm **
Leader/N gene junction	13,001	13,109
N gene/X gene	11,388	11,493
X gene/P gene	11,017	11,122
P gene/Y (MP) gene	9968	9981
Y (MP) gene/M gene	8943	8948
M gene/G gene	8029	8037
G gene/L gene	6038	6043

* Total genome size—13,187 nt; ** Total genome size—13,307 nt.

**Table 2:** Pairwise nucleotide sequence comparisons of RU32-Alm genomic RNA with some other related PhCMoV strains.

PhCMoV Strains *	E-Value	Percentage of Identity	NCBI Accession
**isolate GBVC_PhCMoV_02_IB**	**0.0**	**97.90%**	**MW834321**
**isolate DSMZ PV-1277**	**0.0**	**97.86%**	**ON013917**
**isolate NPPO-NL33372101**	**0.0**	**97.89%**	**OK646029**
**isolate NPPO-NL33226137**	**0.0**	**96.71%**	**OK646028**
**isolate Fr_SM4**	**0.0**	**96.22%**	**MZ574104**
isolate PV-1182	0.0	95.42%	NC055466
isolate Be_GP1	0.0	95.40%	MZ574099
isolate 3216	0.0	95.40%	OQ689795
isolate 232–12	0.0	95.36%	MT269810

* Isolates forming a cluster of X proteins with RU32-Alm are in bold and underlined.

**Table 3:** Pairwise nucleotide sequence comparisons of genomic RNAs from Slovenian isolates with some other related PhCMoV strains.

PhCMoV Strains *	E-Value	Percentage of Identity	NCBI Accession
**isolate BER19ST2**	**0.0**	**100.00%**	**MW366749**
**isolate TUR20ST2**	**0.0**	**97.67%**	**OL472124**
**isolate TUR20ST3**	**0.0**	**97.67%**	**OL472110**
**isolate PLE20ST2**	**0.0**	**97.63%**	**OL472115**
isolate Be_GP1	0.0	96.41%	MZ574099
isolate GH24	0.0	96.43%	OQ689794
isolate 3216	0.0	95.40%	OQ689795

* Isolates forming a cluster of X proteins (Slovenian strains) are shown in bold and underlined.

## Data Availability

The data supporting the findings of this study are available within the article and in the NCBI database, BioProject accession number PRJNA1144656, GenBank accession numbers PQ206275 and PQ206276.

## Supplementary Materials

The following supporting information can be downloaded at https://www.mdpi.com/article/10.3390/v16101538/s1. Figure S1: The phylogeny of the PhCMoV strains based on N (nucleocapsid) protein sequences.
