# Supplementary material for: Genomes of Alphanucleorhabdovirus Physostegiae Isolates from Two Different Cultivar Groups of Solanum melongena
Source: Viruses. 2024 Sep 28;16(10):1538. doi: 10.3390/v16101538 (PMC11512384; doi:10.3390/v16101538)
Supplement: Supplementary file 1 [file viruses-16-01538-s001.zip › Figure S1.pdf]

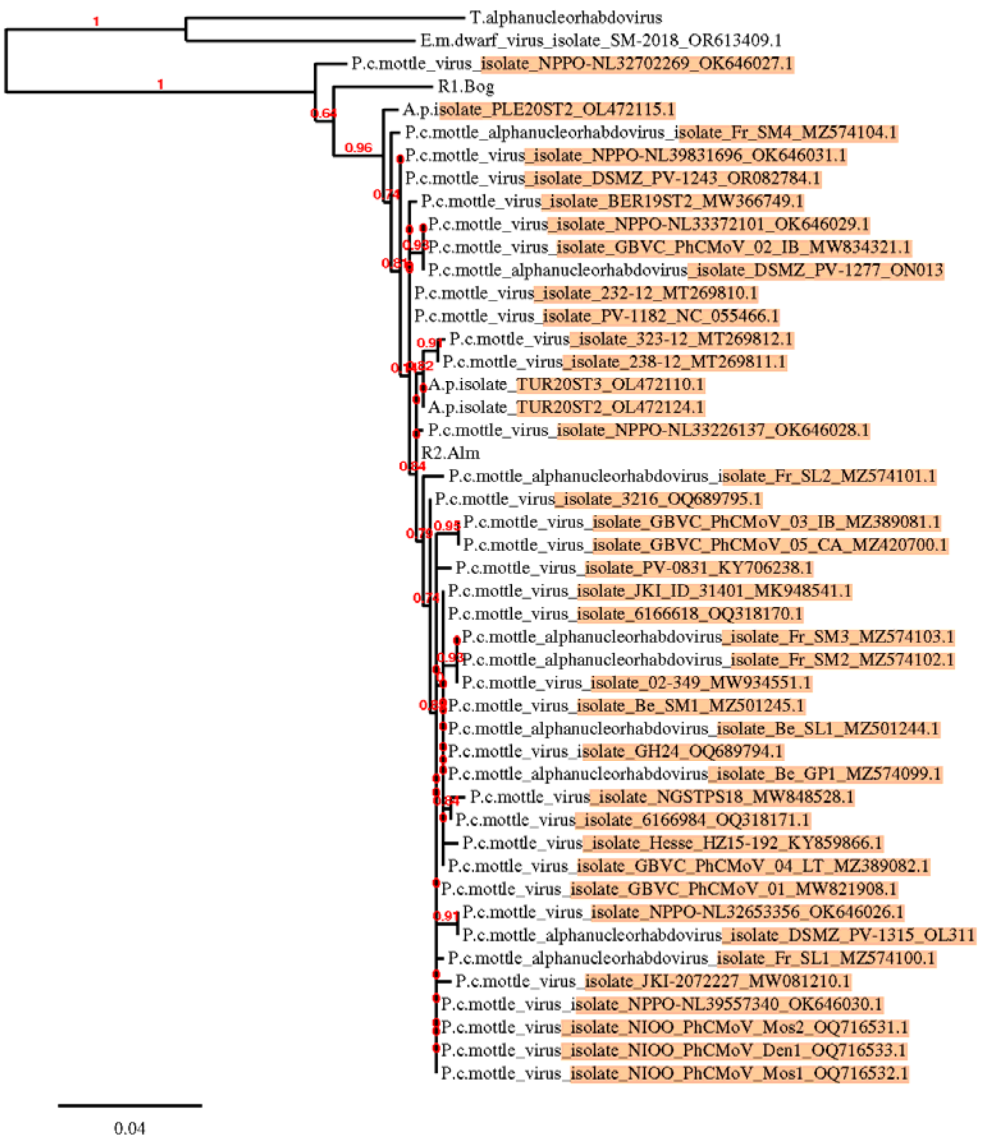


**Figure S1.** Phylogeny of the PhCMoV strains (isolates) based on N (nucleocapsid) protein sequences. Accession numbers are shown. The scale bar indicates the number of substitutions per site.
